# Cross‐Informant Comparison of Depressive Symptoms in Youth: A Network Approach

**DOI:** 10.1002/pchj.70050

**Published:** 2025-09-02

**Authors:** Feifei Chen, Xinlu Sun, Ting Yuan, Xiangjuan Tian, Xinying Li, Nengzhi Jiang

**Affiliations:** ^1^ School of Psychology Shandong Second Medical University Weifang China; ^2^ CAS Key Laboratory of Mental Health, Institute of Psychology Chinese Academy of Sciences Beijing China; ^3^ Department of Psychology University of Chinese Academy of Sciences Beijing China

**Keywords:** cross‐informant discrepancies, depressive symptoms, network analysis, youth

## Abstract

Developmental researchers generally use a multi‐informant approach to assess youth depressive symptoms to increase diagnostic accuracy and reliability, but informant discrepancies between youth and caregivers are common. Previous studies have predominantly used the sum score‐level approach to examine informant discrepancies, which may obscure the heterogeneity of depression. This study adopted a symptom‐level approach, network analysis, to examine informant discrepancies regarding depressive symptoms. The participant sample comprised 1043 community youth living in China (*M*_age_ = 13.68, 48.3% male) and their caregivers. Youth and caregivers completed the Children's Depression Inventory‐Youth (CDI‐Y) and the Children's Depression Inventory‐Parents (CDI‐P) separately. We employed R 4.3.0 and the Ising model to estimate two distinct networks. We then utilized the R‐package Network Comparison Test to compare these two networks. Our findings revealed that irritability emerged as a symptom with high centrality in both networks, while crying demonstrated the most significant disparity in strength centrality, being stronger in the youth‐report network. Youth‐reported crying showed stronger connections with suicidal ideation (edge weight = 2.78), social withdrawal (edge weight = 1.72) and schoolwork difficulty (edge weight = 1.70), whereas caregivers‐reported crying was more strongly associated with self‐hatred (edge weight = 1.21). This study contributes to a better understanding of the structure of depressive symptoms from the perspectives of both youth and their caregivers.

## 1 Introduction

Major depressive disorder (MDD) is the most prevalent mental disorder among adolescents worldwide, and its prevalence has been rising over the past decades (Twenge et al. 2019). Studies suggest that, even among adolescents who do not meet the criteria for MDD, the presence of depressive symptoms is associated with a variety of negative outcomes. According to a meta‐analysis, the prevalence of depressive symptoms among middle school students in China was found to be 24.3% (Tang et al. 2019). Depressive symptoms increase the likelihood of suicidal thoughts (Zubrick et al. 2017), functional impairment (Schuler et al. 2021), and reduced academic performance (Yong et al. 2022), and are associated with poor social and health outcomes in adulthood (Naicker et al. 2013).

Adolescence is a dynamic period marked by heightened emotional instability—including increased mood fluctuations and irritability—as well as evolving social relationships (Bailen et al. 2019; Halyna and Lyubov 2021). During this stage, adolescents increasingly seek emotional support from peers rather than parents and develop a stronger need for autonomy and privacy (Goossens 2018; Laursen and Hartl 2013). These emotional and relational changes may limit parental awareness of internalizing symptoms and contribute to informant discrepancies in depression assessments.

The majority of studies recommend adopting a multi‐informant approach when assessing depressive symptoms in adolescents, encompassing the perspectives of the adolescents themselves, as well as their caregivers, teachers, and doctors (Klein et al. 2005). Caregivers in particular play an important role as informants. They serve as a source of information, often disclosing details that adolescents may hesitate to report. However, caregivers can also influence adolescents' decisions regarding seeking medical attention and can impact the onset of adolescent depression (Angold et al. 2006). Although obtaining data from multiple informants is considered optimal, it is crucial to acknowledge that there are moderated agreement between these informants and cross‐informant discrepancies (De Los Reyes et al. 2015). This cross‐informant discrepancy can affect the caregiver‐youth relationship quality and the development of adolescent psychopathology (Laird and De Los Reyes 2012). In clinical practice, it can be challenging to decide which treatments should be prioritized if there are cross‐informant discrepancies (De Los Reyes and Kazdin 2005). Still, some researchers have pointed out that if data from multiple informants point to the same conclusion, the research results should be considered to be more accurate and valuable (De Los Reyes et al. 2019). Overall, advancing research on the consistency and heterogeneity of adolescent depressive symptoms reported by multiple informants can help alleviate the burden of depression in adolescents and improve clinical diagnosis and treatment processes.

The majority of studies focusing on the consistency and heterogeneity of depression reported by youth and caregivers are based on the sum score‐level approach. This approach involves the comparison of informant discrepancies using methods such as calculating correlations, standardized difference scores, or regression‐based analyses. Existing research yields conflicting results regarding the reporting of depressive symptoms, with some studies indicating that parents report more depressive symptoms compared to the self‐reports provided by adolescents, while other studies have found an opposite trend (Eg et al. 2018). In general population samples, children tend to report higher depression severity than their parents (Martinuzzi et al. 2018). However, it is worth noting that adolescent depression is a very heterogeneous syndrome, and the sum score‐level approach may mask the contributions of individual symptoms and the variations in associations among depressive symptoms (Borsboom 2008; Borsboom et al. 2003; Fried and Nesse 2015).

Network theory conceptualizes psychiatric disorders as causal systems composed of interacting symptoms (Borsboom 2017; Borsboom and Cramer 2013). Within network theory, symptoms are referred to as nodes, and the associations between symptoms are referred to as edges. Network analysis addresses the limitations of traditional sum‐score methods by enabling examination at the level of individual symptoms. Specifically, network analysis has the following advantages: First, it employs centrality indices (e.g., strength) to quantify the importance of each symptom within the network. Symptoms with higher centrality—often referred to as “central symptoms”—may serve as more effective intervention targets (Beard et al. 2016; Borsboom and Cramer 2013). Targeting these symptoms could produce a cascading effect, leading to a more efficient alleviation of overall symptom severity (Beard et al. 2016). Second, it allows for the visualization and analysis of the pattern of associations among symptoms (edges). Stronger inter‐symptom connections imply higher global strength, which indicates a more sensitive network that is more susceptible to external stimuli and thus more prone to developing into a pathological state (van Borkulo and Millner 2016). Third, network analysis facilitates comparison between different networks with the same set of symptoms, enabling assessment of differences in centrality and overall structure (van Borkulo and Millner 2016). Therefore, network analysis holds the potential to enhance our comprehension of multi‐informant discrepancies.

Network analysis has only recently begun to receive attention in multi‐informant discrepancies research, and only two studies thus far have employed network analysis methods to investigate multi‐informant discrepancies in the reporting of depressive symptoms. One study compared the networks reported by depression patients (*M*_age_ = 50.2) and doctors and found that the connection between guilt and suicidal ideation was stronger in the network reported by patients (Feiten et al. 2021). The other study focused on the disparities in symptom networks reported by depressed children (*M*_age_ = 7.8) and their mothers. In this study, sadness emerged as the symptom with the highest strength centrality in both networks; however, differences were observed in the patterns of associations among depressive symptoms (Seneldir et al. 2022). These investigations have contributed significantly to our understanding of informant discrepancies, offering valuable insights for clinical diagnosis and treatment. However, researchers and clinicians have voiced concern about the use of different measures across subjects and have recommended that future studies consider utilizing both the child and parent versions of the Children's Depression Inventory (CDI) together, with the aim of mitigating the potential impact of item wording and other scale‐related differences (Seneldir et al. 2022). Furthermore, previous studies have focused on children and middle‐aged adults, but adolescence is a critical stage during which individuals are more susceptible to mental health problems and thus deserves particular attention in both research and clinical practice (Dahl et al. 2018; Feiten et al. 2021; Seneldir et al. 2022).

Therefore, this study employed network analysis to investigate informant discrepancies and consistencies between youth‐ and caregiver‐rated depressive symptoms networks. We hypothesized that (1) core emotional symptoms of depression (e.g., irritability, sadness) would demonstrate high centrality and consistency across both youth‐ and caregiver‐rated networks, given their pervasive role in depressive psychopathology; whereas (2) behavioral symptoms with context‐dependent expression (e.g., crying, disobedience) would exhibit notable informant discrepancies, reflecting differences in symptom visibility or reporting biases between adolescents and caregivers.

## 2 Materials and Methods

### 2.1 Participants

This cross‐sectional study employed a cluster convenience sampling method to recruit 1303 Chinese adolescents aged 8–17 years (*M* = 13.68, SD = 1.32; 549 females) and their primary caregivers (211 fathers, 832 mothers) in Beijing. To ensure data quality, we implemented a three‐step screening process: (1) excluded cases with > 15% missing variables (*n* = 44); (2) removed non‐serious responses (e.g., straight‐lining; *n* = 44); and (3) eliminated multivariate outliers using Mahalanobis distance (*χ* ^2^ = 55.48, *p* < 0.001; *n* = 172). The final analytical sample comprised 1043 dyads, with no significant differences in age, gender, or family income between excluded (*n* = 260) and included participants (*p* > 0.05), suggesting random missingness.

The sample included 350 elementary school, 434 junior high school, and 259 senior high school students. Caregivers averaged 41.17 years old (SD = 9.71), with 70.1% of fathers and 60.2% of mothers having completed high school or higher education. Household monthly income exceeded ¥5000 in 75.9% of families. The study was approved by the Institutional Review Board of the corresponding author's university (Protocol: 2021YX027) and conducted in accordance with the Declaration of Helsinki. Written informed consent was obtained from all participants and their guardians, with clear instructions about voluntary participation and withdrawal rights.

### 2.2 Measurements

The Chinese version of the CDI was used to measure depressive symptoms in the current study (Kovacs 1992). Both the youth‐report version (Children's Depression Inventory‐Youth; CDI‐Y) and the parent report version (Children's Depression Inventory‐Parent; CDI‐P) contain identical items, with the primary distinction being that caregivers are asked to report how they believe their child feels (Wierzbicki 1987). Each version comprises 27 items rated on a 3‐point Likert scale ranging from 1 (“*I do most things OK*”; “*My child does most things OK*”) to 3 (“*I do everything wrong*”; “*My child does everything wrong*”). Youth participants rated their own depressive symptoms over the past 2 weeks, while caregivers rated their perception of their child's symptoms over the same time frame. The Chinese version of CDI‐Y has demonstrated satisfactory structural validity and robust psychometric properties, with internal consistency reported at *α* = 0.85 and test–retest reliability at *r* = 0.75 (Yu and Li 2000). Although there are currently no published validation studies specifically examining the Chinese version of the CDI‐P, prior international research indicates that CDI‐P items are directly derived from the CDI‐Y and exhibit comparable psychometric properties, including internal consistency (*α* = 0.74) and test–retest reliability (*r* = 0.75) (Wierzbicki 1987). In our sample, both the CDI‐Y (*α* = 0.792) and CDI‐P (*α* = 0.808) demonstrated excellent internal consistency.

### 2.3 Statistical Analysis

All data analysis was performed using R 4.3.0.

#### 2.3.1 Exploratory Analysis

We initiated the data preparation process by utilizing the “merge” function to identify which participants had completed both the CDI‐Y and the CDI‐P. An assessment of missing data was conducted, and participants with missing values exceeding 15% were excluded from the dataset. To address any other missing data, we employed the *mice* package to implement multiple imputation techniques (van Buuren et al. 2020; Zhang 2016). The *careless* package was then utilized to identify and exclude participants who may not have provided serious responses. Due to the substantial skewness and kurtosis observed in our research variables, we opted to binarize the variable values (Van Borkulo et al. 2014). The values of all CDI‐Y and CDI‐P items were set to either 1 or 0, with a 1 (item value was 2 or 3) indicating symptom occurrence and a 0 (item value was 1) indicating symptom absence (Fried and Nesse 2015).

#### 2.3.2 Network Estimation

We employed the “goldbricker” function (with a threshold of 0.20 at *p* = 0.05) to examine potential item redundancy in our psychometric network. The analysis confirmed that all CDI‐Y and CDI‐P items measure distinct psychological constructs, as none showed statistically significant overlap (Tables S1 and S2). Consequently, we retained all 27 items in our network analysis.

We used the *bootnet* package and the “estimateNetwork” function to estimate an unregularized Ising model for depressive symptoms as reported by caregivers and youths (Van Borkulo et al. 2014). To visualize the entire network, we used the *qgraph* package (Epskamp et al. 2012).

#### 2.3.3 Node Centrality

We used the *qgraph* package and the “centralityPlot” function to visualize the centrality of nodes. Node centrality indicates the importance of nodes in the network. Symptoms with high centrality are referred to as central symptoms. When a central symptom is activated, its associated symptoms may also be activated. Node centrality metrics include strength, closeness, and betweenness (Valente 2012). Strength is the sum of the edge weights connected to a node; as this is considered to be the most stable and reliable measure of node centrality, this study focused on strength centrality (Epskamp et al. 2018). We were interested in which node was central in both networks (i.e., stronger than at least 50% of the other nodes within the network) and which node had the greatest difference in centrality between the two networks.

#### 2.3.4 Network Accuracy

A case‐dropping subset bootstrap was performed 1000 times using the *bootnet* package, and the centrality‐stability coefficient (CS‐coefficient) was calculated to examine the stability of the centrality metrics (Newman and Girvan 2004). The CS‐coefficient represents the maximum proportion of cases that can be dropped to still obtain a 95% probability that the correlation of the ranking between the original network and the case‐subset network will amount to 0.7. It was suggested that the CS‐coefficient should be at least greater than 0.25, and preferably greater than 0.5, as a larger CS‐coefficient indicates a more stable network (Newman and Girvan 2004). To check the accuracy of edge weights, we calculated 95% confidence intervals (CIs) for edges using non‐parametric bootstrapping using 1000 samples; narrower CIs indicate more accurate edges (Newman and Girvan 2004).

#### 2.3.5 Network Comparison Test

We used the *NetworkComparisonTest* package to compare the differences between the CDI‐Y and CDI‐P networks (van Borkulo and Millner 2016). Specifically, we used a 1000 permutation‐based hypothesis test to examine the two networks in terms of node centrality, edge strength, global strength, and network structure. Global strength was the sum of absolute weights of all edges in the network, and it indicates the degree of network connectivity (Opsahl et al. 2010). The higher value of global strength indicated more likely to develop mental disorders (Van Borkulo et al. 2022). The network structure focuses on the edge with the greatest difference between the two networks.

## 3 Results

### 3.1 Descriptive Statistics

Table 1 presents the prevalence, skewness, kurtosis, means, standard deviations, and paired‐sample *t*‐test results for depressive symptoms reported by 1043 youths. Significant discrepancies were found for 24 out of 27 symptoms (88.89%), indicating systematic differences between youth and caregiver reports. Youths reported higher symptom severity than caregivers on 22 items, while caregivers reported greater severity on 2 items (CDI21: School Dislike; CDI22: Lack of Friendship). No significant discrepancies were observed for three symptoms: CDI4 (Anhedonia), CDI11 (Irritability), and CDI20 (Loneliness). Indecisiveness (CDI13) showed the highest observed mean score and prevalence among youth‐reported symptoms, while School Dislike (CDI21) showed the highest observed mean score and prevalence among caregivers‐reported symptoms, although no formal statistical comparisons across items were conducted. The mean total score for youth‐reported depressive symptoms was significantly higher than that reported by caregivers (*M*_youth_ = 1.47, *M*_caregivers_ = 1.27, *t* = 26.03, *p* < 0.001).

**TABLE 1:** Mean, standard deviation, skewness, kurtosis, and frequency of cdi symptoms (*N* = 1043).

CDI	Symptoms	% Presence (1)		% Absence (0)		Skewness		Kurtosis		Mean		Standard Deviation		*t*
		CDI‐Y	CDI‐P	CDI‐Y	CDI‐P	CDI‐Y	CDI‐P	CDI‐Y	CDI‐P	CDI‐Y	CDI‐P	CDI‐Y	CDI‐P	
1	Sadness	26.37	14.08	73.63	85.92	1.55	2.49	1.49	5.62	1.30	1.15	0.52	0.39	10.89****
2	Pessimism	40.00	24.48	60.00	75.52	0.99	1.74	−0.12	1.92	1.48	1.31	0.64	0.58	12.13***
3	Self‐deprecation	18.30	9.51	81.70	90.49	1.93	3.27	2.73	10.68	1.19	1.10	0.42	0.33	10.83***
4	Anhedonia	31.12	30.85	68.88	69.15	1.21	1.04	0.44	−0.31	1.34	1.32	0.52	0.49	0.90
5	Misbehavior	13.90	11.84	86.10	88.16	2.69	2.95	6.75	8.49	1.16	1.13	0.43	0.39	5.49***
6	Pessimistic worrying	36.59	12.65	63.41	87.35	1.04	2.86	0.08	7.82	1.41	1.15	0.57	0.41	18.52***
7	Self‐hatred	13.54	5.29	86.46	94.71	2.73	4.86	7.02	25.01	1.16	1.06	0.42	0.28	11.15***
8	Self‐blame	39.10	20.81	60.90	79.19	1.02	2.00	−0.03	3.11	1.46	1.25	0.62	0.52	11.28***
9	Suicidality	49.24	7.89	50.76	92.11	0.50	3.60	−1.40	13.12	1.75	1.08	0.84	0.30	34.34***
10	Crying	42.15	2.87	57.85	97.13	0.55	6.77	−1.55	48.28	1.73	1.04	0.90	0.23	33.39***
11	Irritability	27.71	27.35	72.29	72.65	1.52	1.47	1.00	0.66	1.37	1.39	0.65	0.69	1.39
12	Social withdrawal	42.60	16.05	57.40	83.95	0.60	2.43	−1.44	4.85	1.71	1.21	0.88	0.52	23.60***
13	Indecisiveness	68.79	45.20	31.21	54.80	−0.26	0.77	−1.60	−0.81	2.14	1.61	0.86	0.74	22.74***
14	Negative body image	59.37	35.70	40.63	64.30	0.33	0.84	−1.25	−0.62	1.81	1.37	0.77	0.51	21.62***
15	School work difficulty	60.63	31.03	39.37	68.97	0.16	1.29	−1.54	0.14	1.91	1.44	0.83	0.72	22.26***
16	Sleep disturbance	23.50	11.57	76.50	88.43	1.81	3.06	2.24	8.98	1.29	1.14	0.56	0.41	9.75***
17	Fatigue	22.87	12.38	77.13	87.62	1.85	2.91	2.31	7.75	1.29	1.16	0.58	0.46	9.48***
18	Reduced appetite	34.62	22.33	65.38	77.67	1.21	1.85	0.27	2.57	1.43	1.26	0.65	0.51	11.51***
19	Somatic concerns	26.73	15.25	73.27	84.75	1.58	2.51	1.55	5.74	1.31	1.18	0.55	0.44	9.51***
20	Loneliness	18.03	14.89	81.97	85.11	2.23	2.35	4.24	4.84	1.21	1.16	0.48	0.39	3.72
21	School dislike	51.30	56.68	48.70	43.32	0.57	0.43	−0.60	−0.67	1.58	1.65	0.62	0.63	−3.93***
22	Lack of friendship	45.11	57.04	54.89	42.96	0.45	0.16	−1.13	−0.92	1.47	1.60	0.53	0.55	−8.37***
23	School performance decrement	54.44	49.33	45.56	50.67	0.53	0.68	−0.74	−0.60	1.65	1.59	0.67	0.66	5.78***
24	Low self‐esteem	56.14	37.58	43.86	62.42	0.33	1.04	−0.72	0.08	1.61	1.43	0.58	0.59	11.93***
25	Feeling unloved	53.00	24.13	47.00	75.87	0.37	1.68	−0.78	1.93	1.57	1.27	0.57	0.50	21.53***
26	Disobedience	24.39	14.71	75.61	85.29	1.53	2.40	1.30	5.12	1.26	1.16	0.47	0.39	8.89***
27	Fights	9.96	6.73	90.04	93.27	3.24	4.24	10.53	18.70	1.11	1.08	0.35	0.31	5.41***

*** *p* < 0.001.

### 3.2 Symptom Networks

**FIGURE 1:**
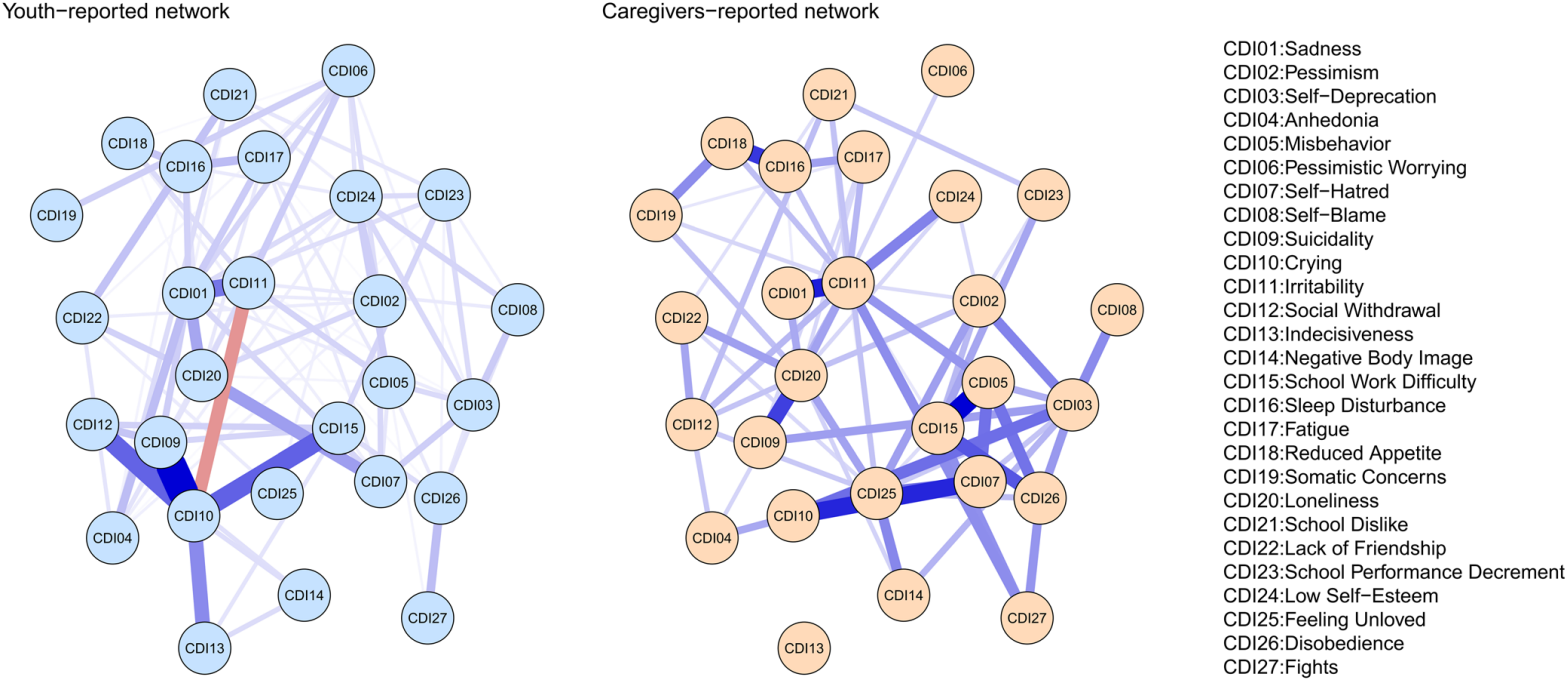
Comparison of depressive symptom networks as reported by youth (left) and caregivers (right). Note. Nodes represent depressive symptoms and edges represent partial correlations between symptoms. Blue and red edges represent positive and negative associations, respectively. Line thickness reflects association strength. The stronger and more saturated edges represent stronger, regularized partial correlations.

Figure 1 displays the network comparison of depressive symptoms as reported by youth (CDI‐Y; left panel) and caregivers (CDI‐P; right panel).

The CDI‐Y network comprised 89 statistically significant edges, with only one negative association. The strongest associations were observed between suicidal ideation and crying (*edge weight = 2.78*), followed by crying and social withdrawal (*edge weight = 1.72*), crying and school work difficulty (*edge weight = 1.70*), and crying and indecisiveness (*edge weight = 1.50*, see Figure S1).

The CDI‐P network comprised 69 statistically significant edges, all of which were positive. The most robust associations were between misbehavior and school work difficulty (*edge weight = 1.42*), followed by sadness and irritability (*edge weight = 1.26*), self‐hatred and crying (*edge weight = 1.21*, see Figure S2).

### 3.3 Network Centrality

**FIGURE 2:**
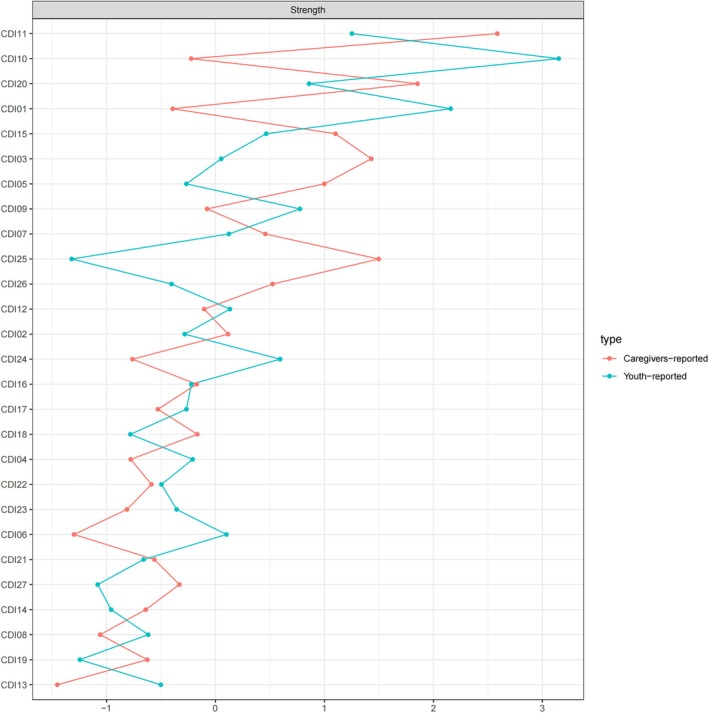
Strength centrality indices comparison (*z*‐scores) between youth‐ and caregivers‐reported networks. Note. Higher values of strength reflect greater node centrality. CDI01 = Sadness; CDI02 = Pessimism; CDI03 = Self‐Deprecation; CDI04 = Anhedonia; CDI05 = Misbehavior; CDI06 = Pessimistic Worrying; CDI07 = Self‐Hatred; CDI08 = Self‐Blame; CDI09 = Suicidal Ideation; CDI10 = Crying; CDI11 = Irritability; CDI12 = Social Withdrawal; CDI13 = Indecisiveness; CDI14 = Negative Body Image; CDI15 = School Work Difficulty; CDI16 = Sleep Disturbance; CDI17 = Fatigue; CDI18 = Reduced Appetite; CDI19 = Somatic Concerns; CDI20 = Loneliness; CDI21 = School Dislike; CDI22 = Lack of Friendship; CDI23 = School Performance Decrement; CDI24 = Low Self‐Esteem; CDI25 = Feeling Unloved; CDI26 = Disobedience; CDI27 = Fights.

Figure 2 depicts the network centrality indices for the CDI‐Y and CDI‐P networks. In the CDI‐Y network, crying showed the highest strength centrality (CDI10: Strength = 9.20) among all symptoms based on observed values. Sadness (CDI01: Strength = 5.50), irritability (CDI11: Strength = 5.40), and suicidal ideation (CDI09: Strength = 4.50) demonstrated relatively high observed centrality values (see Figure S3).

In the CDI‐P network, irritability *showed the highest strength centrality* (CDI11: *Strength = 6.60*) among all symptoms based on observed values. Loneliness (CDI20: *Strength = 5.40*), feeling unloved (CDI25: *Strength = 4.80*), and self‐deprecation (CDI03: *Strength = 4.70*) also demonstrated relatively high observed centrality values (see Figure S4).

### 3.4 Network Stability and Accuracy

Network stability analyses revealed distinct robustness patterns between the CDI‐Y and CDI‐P networks.

For the CDI‐Y network, strength centrality demonstrated excellent stability (CS‐coefficient = 0.67, see Figure S5), indicating that 67% of the sample could be excluded via case‐dropping bootstrap analyses while maintaining strong rank‐order preservation in symptom centrality (Spearman's *ρ* = 0.70). This high level of stability was supported by adequate accuracy across all 89 edges, as evidenced by narrow nonparametric bootstrap confidence intervals (see Figure S6).

In contrast, the CDI‐P network exhibited moderate strength stability (CS‐coefficient = 0.28, see Figure S7). In addition, the precision of edge weight estimates remained acceptable, with similarly constrained confidence intervals across all 69 edges (see Figure S8). Both networks met recommended psychometric standards for interpretability in network models (Epskamp et al. 2018), supporting the robustness of our findings.

### 3.5 Network Comparison Test Results

The results of the network comparison test showed that there was a significant difference in the structure of the depression symptoms networks reported by youth and their caregivers (maximum difference = 2.92; *p* < 0.001), as well as a significant difference in their global strength (global strength difference = 11.59; CDI‐Y global strength = 37.71, CDI‐P global strength = 26.12; *p* = 0.018). The edge with the greatest difference between the two networks was between crying (CDI10) and suicidal ideation (CDI09), which was stronger in the youth‐reported network (see Figures S1 and S2). The node with the greatest difference between the two networks was crying (CDI10), which was stronger in the youth‐reported network (see Figures S3 and S4). However, it should be noted that all reported *p*‐values are uncorrected for multiple comparisons and should be interpreted as exploratory findings to inform future research.

## 4 Discussion

To the best of our knowledge, the present study is the first to use network analysis to examine informant consistencies and discrepancies between depressive symptoms rated by community youths and their caregivers. Our findings reveal that irritability exhibited high centrality and high consistency in both networks. Crying exhibited the most pronounced observed difference in strength centrality and symptom association patterns between the two networks. Youth exhibited strong associations between crying and suicidal ideation, irritability, social withdrawal, and school work difficulty, while caregivers exhibited the strongest associations between crying and self‐hatred.

Irritability was the symptom with the highest consistency and centrality in both networks, which means that irritability is most likely to affect or be affected by the other symptoms. In the diagnosis of depression in children and adolescents, irritability and sadness are considered key symptoms and equally important (Zhou et al. 2022). Furthermore, it has been discovered that irritability is a strong predictor of future depression and serves as a bridge node between depression and other mental disorders (Chen et al. 2022; Jin et al. 2022; Vidal‐Ribas et al. 2016). Currently, there is no reliable method for combining the perspectives of caregivers and youth. Existing research has traditionally relied on degrees of consistency and correlation coefficients to assess the relationship between caregiver and youth assessments. However, when quantifying agreement among informants with correlations, item‐specific agreements or disagreements within an instrument tend to be largely overlooked. In our investigation, we utilized network analysis to evaluate item‐specific cross‐informant compatibility, and identified irritability as the symptom with the highest consistency. Previous studies have found that information discrepancies between different reporters may hinder the enthusiasm and effort of the subjects involved in therapy (e.g., parents, children, therapists) to cooperate to achieve treatment goals, thus affecting the treatment process and outcomes (Yeh and Weisz 2001). Given its high consistency and centrality within both networks, irritability can be considered a promising target for early intervention in the prevention of depression in youths.

Crying exhibited the most pronounced observed difference in strength centrality between the two networks and was stronger in the youth‐reported network. In other words, crying is most likely to affect or be affected by other symptoms in the youth‐reported network. This result is partially consistent with those of previous studies, which found that crying was one of the central symptoms of youth depression (Kim et al. 2021). Crying showed lower centrality in the caregiver‐reported network, which may be linked to the characteristics of our participants. Our study focused on adolescents from China, and previous studies have indicated that persons from collectivist cultures tend to be more emotionally restrained (Matsumoto et al. 2008). Studies have shown that parents' awareness of their children's circumstances is affected by their children's willingness to disclose that information (Booth et al. 2023). Adolescence is a unique stage of development during which individuals grow less dependent on their parents, more dependent on themselves and their peers, and become more willing to share and express their thoughts, feelings, and secrets with friends (Goossens 2018; Laursen and Hartl 2013). It is worth mentioning that crying exhibited its strongest association with suicidal ideation in the youth‐reported network, a relationship that did not manifest to the same extent in the caregiver‐reported network. This implies that youths presenting with crying symptoms may have a higher susceptibility to suicidal ideation, which may potentially be overlooked by caregivers.

We found that there were differences in how crying was associated with other symptoms across the two networks. In the youth‐reported networks, crying was positively associated with suicidal ideation, school difficulty, social withdrawal, and indecisiveness but negatively correlated with irritability. Our findings are consistent with previous studies, which have suggested that the daily stressors of adolescents can be divided into interpersonal relationships and achievement events (Carter and Garber 2011). Interpersonal stressors refer to difficulties interacting with others and can include conflict, rejection, and breakups with their friends; achievement stressors include goal‐related failures and disappointments, such as academic performance (Carter and Garber 2011). Due to greater societal expectations for academic success and lower levels of satisfaction from both themselves and their parents, Asian students typically feel higher levels of academic stress than their Western counterparts (Ma et al. 2018; Tan and Yates 2011; Zhang et al. 2021). According to a study in China, adolescents work hard to improve their academic performance, and that if they are unable to satisfy their parents' expectations, they may experience depressive symptoms like worthlessness, hopelessness, and shame (Tang et al. 2020). Furthermore, our findings revealed a pronounced negative correlation between CDI10 crying and CDI11 irritability in the CDI‐Y network, whereas no such correlation was observed in the CDI‐P network. A previous study found that adolescence is a period of significant emotional development, marked by heightened emotional intensity and reactivity (Derryberry and Rothbart 1997). Youths may experience increased difficulty in regulating their emotions, which can manifest as irritability when they are unable to effectively cope with intense feelings like sadness or frustration (Gross 2002). Crying, as a form of emotional expression, may be triggered by these overwhelming emotions, leading to a negative correlation between crying and irritability as they are both expressions of emotional distress. In the caregiver‐reported network, however, crying was associated with self‐hatred. This can be attributed to the fact that caregivers and adolescents perceive problems differently. According to the Attribution Bias Context (ABC) model, cross‐information discrepancies may be related to cognitive biases, attribution differences, and the informant's reporting context (De Los Reyes and Kazdin 2005). On the one hand, caregivers observe their adolescents' psychological and behavioral problems primarily at home and may be less aware of their adolescents' problems at school. On the other hand, caregivers are more likely to attribute adolescents' psychological and behavioral problems to personality, while adolescents are more likely to attribute their problems to the environment in which the problems arise. Our results suggest that caregivers should pay more attention to the difficulties that their teenagers may be experiencing at school and should give their children more positive feedback to cultivate the youth's self‐concept.

The observed cross‐informant discrepancy in the crying symptom highlights important implications for parent–child communication and the development of supportive strategies in everyday contexts. While youth associated crying with internalized experiences such as suicidal ideation, social withdrawal, and academic stress, caregivers primarily linked it to self‐hatred. This divergence may reflect differences in how emotional expressions are perceived and interpreted across generations (De Los Reyes and Kazdin 2005). Crying, as a visible and emotionally salient symptom, might serve as a potential signal of psychological distress that is readily observable by caregivers but often misunderstood in terms of its underlying emotional meaning (Hyson 2004; Zeman et al. 2006). These findings underscore the importance of fostering open parent–child communication to bridge perceptual gaps and enhance caregivers' understanding of their child's emotional experiences (Ackard et al. 2006; Rohner 2004). In everyday settings such as schools and homes, this discrepancy suggests the need for efforts to promote emotional literacy and awareness (Brackett et al. 2011). For instance, parents may benefit from guidance on interpreting crying within the broader context of adolescents' internal struggles, rather than attributing it solely to negative self‐perceptions. Likewise, adolescents may be supported in developing better emotional expression and communication skills to articulate their needs more directly, thereby reducing potential misinterpretations in the parent–child relationship.

The network comparison test showed significant differences in global strength and network structure between the two networks. In network theory, a more connected network may represent a more severe disorder (Borsboom and Cramer 2013). From this perspective, differences in global strength could indicate that youth and their caregivers have different evaluations of the severity of the youth's depressive symptoms and of the disorder itself. Previous findings on multi‐informant depressive symptoms are conflicting, with one study finding a higher global connection strength when the symptoms were rated by the caregivers, while another found that the child‐report network was more strongly connected than the mother‐report network, which is consistent with our findings (Seneldir et al. 2022). The researchers have found that the direction of these informant discrepancies is related to the characteristics of the sample. In clinical samples, parents often report higher levels of depression severity compared to the self‐reports of children (Cleridou et al. 2017; Goodman 1997). In contrast, in general population samples, children tend to report higher depression severity than their parents (Martinuzzi et al. 2018). One previous study found that, in the general population sample, children often reported higher levels of depression than their parents, but in clinical patients, these results were the opposite (Martinuzzi et al. 2018). Additionally, we found that the two networks were significantly different in terms of structure, and the maximum difference was between crying and suicidal ideation, which was stronger in the CDI‐Y network. A strong link between crying and suicidal ideation shows that the two symptoms might strengthen one another, and that interventions that focus on crying could be useful in reducing suicidal ideation. A previous study on the flow network of adolescent suicidal ideation found a strong direct connection between suicidal ideation and sadness, with crying being the symptom most closely related to sadness (Gijzen et al. 2021).

Beyond conventional sum‐score analyses, our network approach offers three key advances in understanding youth depression: First, by modeling symptom‐level dynamics rather than aggregate scores, we identified irritability and crying as central features that may represent optimal intervention targets—a precision unavailable through traditional methods. Second, our analysis revealed fundamentally different symptom networks between informants that would be obscured by total‐score comparison; youth connected crying to school difficulties while caregivers associated it with self‐hatred, suggesting distinct phenomenological experiences. Third, our matched‐measure design (using parallel CDI forms) controls for measurement artifacts that typically confound cross‐informant comparisons, allowing clearer interpretation of genuine perspective differences rather than instrument effects. These findings demonstrate how network analysis moves beyond static symptom counts to reveal dynamic interaction patterns that may better predict clinical trajectories and inform personalized intervention strategies.

There are nonetheless some limitations to our study as well. First, we used cross‐sectional data, which can only explain correlations between symptoms; however, it is not possible to infer the direction of the associations between the symptoms. Future studies should evaluate the longitudinal stability of the differences in depressive symptoms as reported by youth and their caregivers, and the causal relationships between symptoms. Second, our study used community samples; thus, there are limitations in terms of generalizing to clinical populations. Third, the caregivers in our study included both fathers and mothers; future research should compare the network of adolescent depression symptoms reported by fathers and mothers, who may have different perceptions of adolescent mental health problems. Fourth, although the youth and caregiver questionnaires were completed within the same week, slight temporal discrepancies may still exist between informant reports. Future research should aim to standardize simultaneous or same‐day completion to further reduce potential sources of recall or timing‐related bias. Fifth, potential confounders like socioeconomic status were not controlled, which may influence symptom reporting. Future studies should incorporate these variables in network analyses. Finally, as our sample was Chinese, cultural factors may influence symptom presentation (Wilk and Bolton 2002). Cross‐cultural comparisons are needed to identify universal versus culture‐specific symptom networks.

## 5 Conclusions

The present study used network analysis at the symptom level to examine informant consistencies and discrepancies between youth‐ and caregiver‐reported depressive symptoms. Our findings provide insight into these differences and thus contribute to the literature on cross‐informant compatibility. We found that irritability was a common central symptom that can provide recommendations for the selection of future targets for depression prevention interventions in youth. Youth and caregivers had different perceptions of crying and its association with other symptoms. Youth associate crying with difficulties they may experience at school, such as suicidal ideation, schoolwork difficulty, and social withdrawal. In contrast, caregivers associate crying with self‐hatred. Caregivers should pay more attention to the difficulties their children may be experiencing at school and the strong correlation between crying and suicidal ideation. A symptom‐level approach to understanding informant discrepancies does not require abandoning previous methods but rather calls for us to focus on a different level of analysis. Combining multiple methods can help us better understand the structure and implications of informant discrepancies in depressive symptoms.

## Ethics Statement

According to the Declaration of Helsinki, this study was approved by the Ethics Committee of Shandong Second Medical University (Protocol Number: 2021YX027).

## Consent

Informed consent was obtained from all subjects involved in the study.

## Conflicts of Interest

The authors declare no conflicts of interest.

## Supporting information


**Figure S1:** Nonparametric bootstrapped difference test for edge weights in the CDI‐Y network. *Note*: Gray boxes indicate edge weights that do not differ significantly from one another, while black boxes indicate edge weights that do differ significantly. Blue and red boxes on the diagonal correspond to edge weights with positive and negative correlations, respectively. CDI01 = Sadness; CDI02 = Pessimism; CDI03 = Self‐Deprecation; CDI04 = Anhedonia; CDI05 = Misbehavior; CDI06 = Pessimistic Worrying; CDI07 = Self‐Hatred; CDI08 = Self‐Blame; CDI09 = Suicidal Ideation; CDI10 = Crying; CDI11 = Irritability; CDI12 = Social Withdrawal; CDI13 = Indecisiveness; CDI14 = Negative Body Image; CDI15 = School Work Difficulty; CDI16 = Sleep Disturbance; CDI17 = Fatigue; CDI18 = Reduced Appetite; CDI19 = Somatic Concerns; CDI20 = Loneliness; CDI21 = School Dislike; CDI22 = Lack of Friendship; CDI23 = School Performance Decrement; CDI24 = Low Self‐Esteem; CDI25 = Feeling Unloved; CDI26 = Disobedience; CDI27 = Fights.

**Figure S2:** Nonparametric bootstrapped difference test for edge weights in the CDI‐P network. *Note*: Gray boxes indicate edge weights that do not differ significantly from one another, while black boxes indicate edge weights that do differ significantly. Blue and red boxes on the diagonal correspond to edge weights with positive and negative correlations, respectively. CDI01 = Sadness; CDI02 = Pessimism; CDI03 = Self‐Deprecation; CDI04 = Anhedonia; CDI05 = Misbehavior; CDI06 = Pessimistic Worrying; CDI07 = Self‐Hatred; CDI08 = Self‐Blame; CDI09 = Suicidal Ideation; CDI10 = Crying; CDI11 = Irritability; CDI12 = Social Withdrawal; CDI13 = Indecisiveness; CDI14 = Negative Body Image; CDI15 = School Work Difficulty; CDI16 = Sleep Disturbance; CDI17 = Fatigue; CDI18 = Reduced Appetite; CDI19 = Somatic Concerns; CDI20 = Loneliness; CDI21 = School Dislike; CDI22 = Lack of Friendship; CDI23 = School Performance Decrement; CDI24 = Low Self‐Esteem; CDI25 = Feeling Unloved; CDI26 = Disobedience; CDI27 = Fights.

**Figure S3:** Nonparametric bootstrapped difference test for node strength in the CDI‐Y network. *Note*: Gray boxes indicate node strength that do not differ significantly from one another, while black boxes indicate node strength that do differ significantly. The numbers in the white boxes (i.e., diagonal line) represent the values of node strength. CDI01 = Sadness; CDI02 = Pessimism; CDI03 = Self‐Deprecation; CDI04 = Anhedonia; CDI05 = Misbehavior; CDI06 = Pessimistic Worrying; CDI07 = Self‐Hatred; CDI08 = Self‐Blame; CDI09 = Suicidal Ideation; CDI10 = Crying; CDI11 = Irritability; CDI12 = Social Withdrawal; CDI13 = Indecisiveness; CDI14 = Negative Body Image; CDI15 = School Work Difficulty; CDI16 = Sleep Disturbance; CDI17 = Fatigue; CDI18 = Reduced Appetite; CDI19 = Somatic Concerns; CDI20 = Loneliness; CDI21 = School Dislike; CDI22 = Lack of Friendship; CDI23 = School Performance Decrement; CDI24 = Low Self‐Esteem; CDI25 = Feeling Unloved; CDI26 = Disobedience; CDI27 = Fights.

**Figure S4:** Nonparametric bootstrapped difference test for node strength in the CDI‐P network. *Note*: Gray boxes indicate node strength that do not differ significantly from one another, while black boxes indicate node strength that do differ significantly. The numbers in the white boxes (i.e., diagonal line) represent the values of node strength. CDI01 = Sadness; CDI02 = Pessimism; CDI03 = Self‐Deprecation; CDI04 = Anhedonia; CDI05 = Misbehavior; CDI06 = Pessimistic Worrying; CDI07 = Self‐Hatred; CDI08 = Self‐Blame; CDI09 = Suicidal Ideation; CDI10 = Crying; CDI11 = Irritability; CDI12 = Social Withdrawal; CDI13 = Indecisiveness; CDI14 = Negative Body Image; CDI15 = School Work Difficulty; CDI16 = Sleep Disturbance; CDI17 = Fatigue; CDI18 = Reduced Appetite; CDI19 = Somatic Concerns; CDI20 = Loneliness; CDI21 = School Dislike; CDI22 = Lack of Friendship; CDI23 = School Performance Decrement; CDI24 = Low Self‐Esteem; CDI25 = Feeling Unloved; CDI26 = Disobedience; CDI27 = Fights.

**Figure S5:** Stability of node strength in the CDI‐Y network. *Note*: The *x*‐axis represents the percentage of cases in the original sample used at each step. The *y*‐axis represents the average of correlations between the centrality indices in the original network and the centrality indices in the networks that were re‐estimated after dropping increasing percentages of cases.

**Figure S6:** Accuracy of edge weights in the CDI‐Y network. *Note*: The red line depicts the sample edge weights and the gray bar depicts the bootstrapped confidence interval. CDI01 = Sadness; CDI02 = Pessimism; CDI03 = Self‐Deprecation; CDI04 = Anhedonia; CDI05 = Misbehavior; CDI06 = Pessimistic Worrying; CDI07 = Self‐Hatred; CDI08 = Self‐Blame; CDI09 = Suicidal Ideation; CDI10 = Crying; CDI11 = Irritability; CDI12 = Social Withdrawal; CDI13 = Indecisiveness; CDI14 = Negative Body Image; CDI15 = School Work Difficulty; CDI16 = Sleep Disturbance; CDI17 = Fatigue; CDI18 = Reduced Appetite; CDI19 = Somatic Concerns; CDI20 = Loneliness; CDI21 = School Dislike; CDI22 = Lack of Friendship; CDI23 = School Performance Decrement; CDI24 = Low Self‐Esteem; CDI25 = Feeling Unloved; CDI26 = Disobedience; CDI27 = Fights.

**Figure S7:** Stability of node strength in the CDI‐P network. *Note*: The *x*‐axis represents the percentage of cases in the original sample used at each step. The *y*‐axis represents the average of correlations between the centrality indices in the original network and the centrality indices in the networks that were re‐estimated after dropping increasing percentages of cases.

**Figure S8:** Accuracy of edge weights in the CDI‐P network. *Note*: The red line depicts the sample edge weights and the gray bar depicts the bootstrapped confidence interval. CDI01 = Sadness; CDI02 = Pessimism; CDI03 = Self‐Deprecation; CDI04 = Anhedonia; CDI05 = Misbehavior; CDI06 = Pessimistic Worrying; CDI07 = Self‐Hatred; CDI08 = Self‐Blame; CDI09 = Suicidal Ideation; CDI10 = Crying; CDI11 = Irritability; CDI12 = Social Withdrawal; CDI13 = Indecisiveness; CDI14 = Negative Body Image; CDI15 = School Work Difficulty; CDI16 = Sleep Disturbance; CDI17 = Fatigue; CDI18 = Reduced Appetite; CDI19 = Somatic Concerns; CDI20 = Loneliness; CDI21 = School Dislike; CDI22 = Lack of Friendship; CDI23 = School Performance Decrement; CDI24 = Low Self‐Esteem; CDI25 = Feeling Unloved; CDI26 = Disobedience; CDI27 = Fights.

**Table S1:** Proportional distribution of significantly different correlations among symptom pairs in the CDI‐Y.

**Table S2:** Proportional distribution of significantly different correlations among symptom pairs in the CDI‐P scale.

## Data Availability

The data that support the findings of this study are available from the corresponding author upon reasonable request.
