# Supplementary figures and images for: Cross‐Informant Comparison of Depressive Symptoms in Youth: A Network Approach
Source: Psych J. 2025 Sep 2;14(5):685–96. doi: 10.1002/pchj.70050 (PMC12520835; doi:10.1002/pchj.70050)

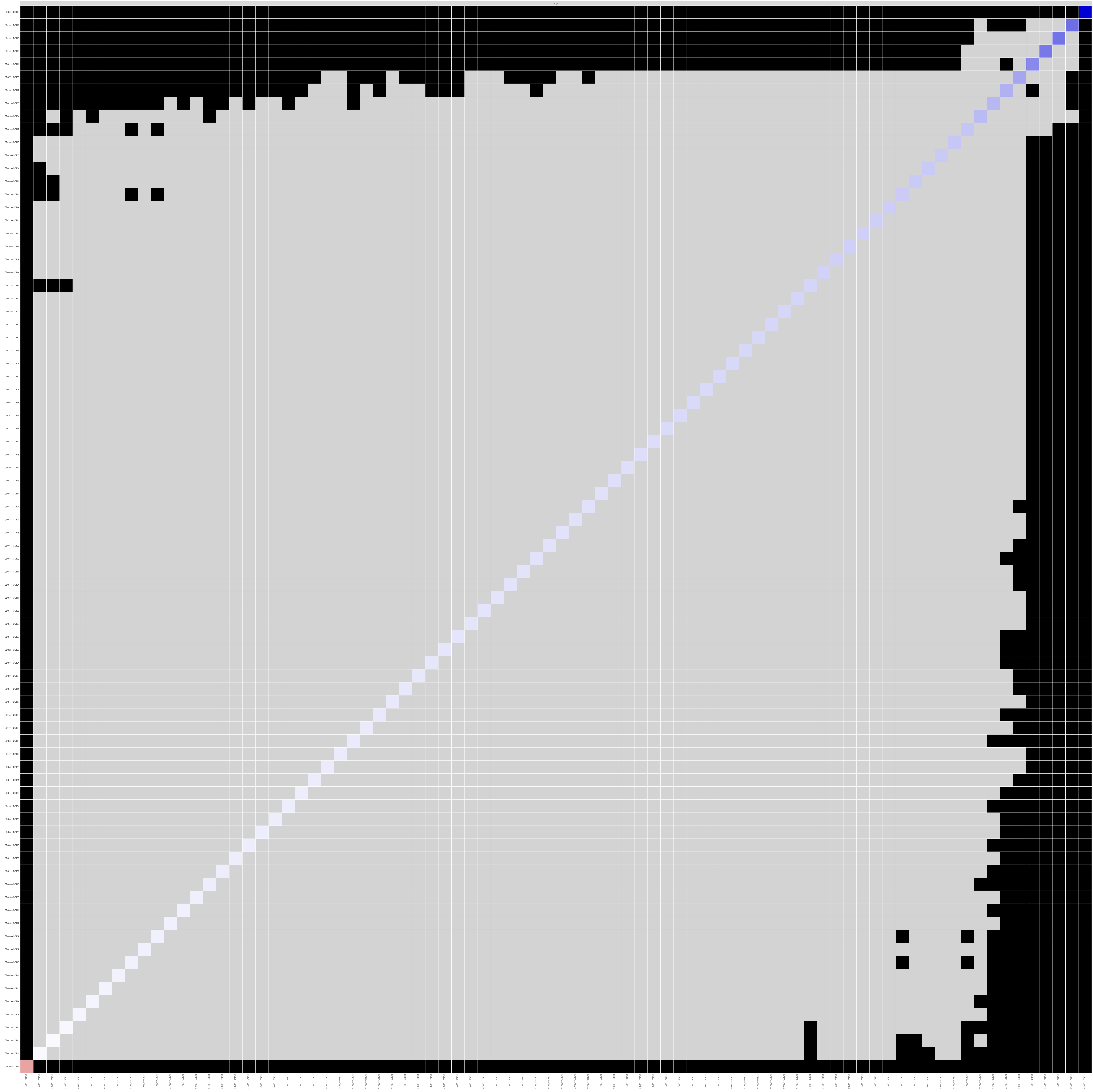

Supplement: Supplementary file 1 — Figure S1: Nonparametric bootstrapped difference test for edge weights in the CDI‐Y network. Note: Gray boxes indicate edge weights that do not differ significantly from one another, while black boxes indicate edge weights that do differ significantly. Blue and red boxes on the diagonal correspond to edge weights with positive and negative correlations, respectively. CDI01 = Sadness; CDI02 = Pessimism; CDI03 = Self‐Deprecation; CDI04 = Anhedonia; CDI05 = Misbehavior; CDI06 = Pessimistic Worrying; CDI07 = Self‐Hatred; CDI08 = Self‐Blame; CDI09 = Suicidal Ideation; CDI10 = Crying; CDI11 = Irritability; CDI12 = Social Withdrawal; CDI13 = Indecisiveness; CDI14 = Negative Body Image; CDI15 = School Work Difficulty; CDI16 = Sleep Disturbance; CDI17 = Fatigue; CDI18 = Reduced Appetite; CDI19 = Somatic Concerns; CDI20 = Loneliness; CDI21 = School Dislike; CDI22 = Lack of Friendship; CDI23 = School Performance Decrement; CDI24 = Low Self‐Esteem; CDI25 = Feeling Unloved; CDI26 = Disobedience; CDI27 = Fights. [file PCHJ-14-685-s006.pdf]

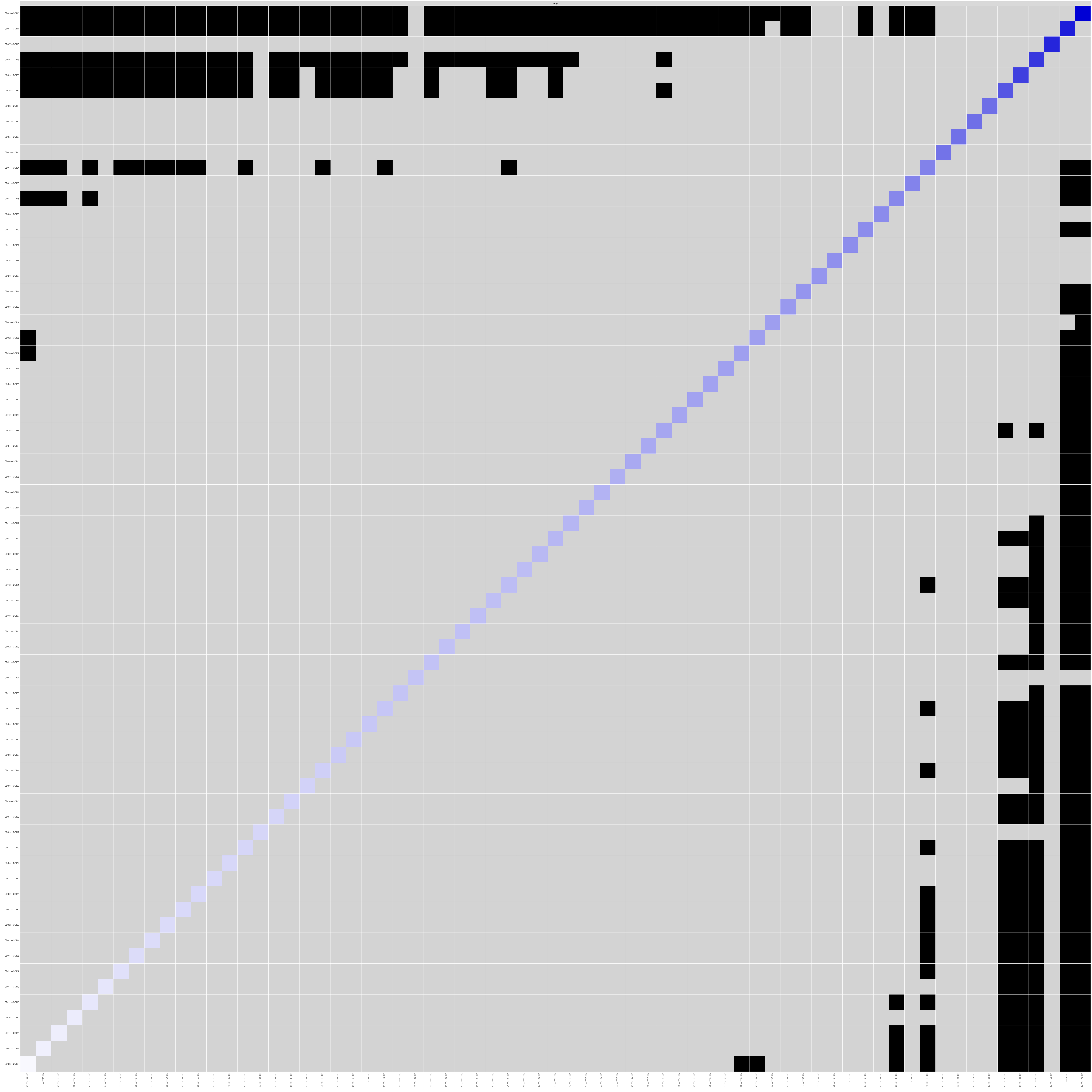

Supplement: Supplementary file 2 — Figure S2: Nonparametric bootstrapped difference test for edge weights in the CDI‐P network. Note: Gray boxes indicate edge weights that do not differ significantly from one another, while black boxes indicate edge weights that do differ significantly. Blue and red boxes on the diagonal correspond to edge weights with positive and negative correlations, respectively. CDI01 = Sadness; CDI02 = Pessimism; CDI03 = Self‐Deprecation; CDI04 = Anhedonia; CDI05 = Misbehavior; CDI06 = Pessimistic Worrying; CDI07 = Self‐Hatred; CDI08 = Self‐Blame; CDI09 = Suicidal Ideation; CDI10 = Crying; CDI11 = Irritability; CDI12 = Social Withdrawal; CDI13 = Indecisiveness; CDI14 = Negative Body Image; CDI15 = School Work Difficulty; CDI16 = Sleep Disturbance; CDI17 = Fatigue; CDI18 = Reduced Appetite; CDI19 = Somatic Concerns; CDI20 = Loneliness; CDI21 = School Dislike; CDI22 = Lack of Friendship; CDI23 = School Performance Decrement; CDI24 = Low Self‐Esteem; CDI25 = Feeling Unloved; CDI26 = Disobedience; CDI27 = Fights. [file PCHJ-14-685-s001.pdf]

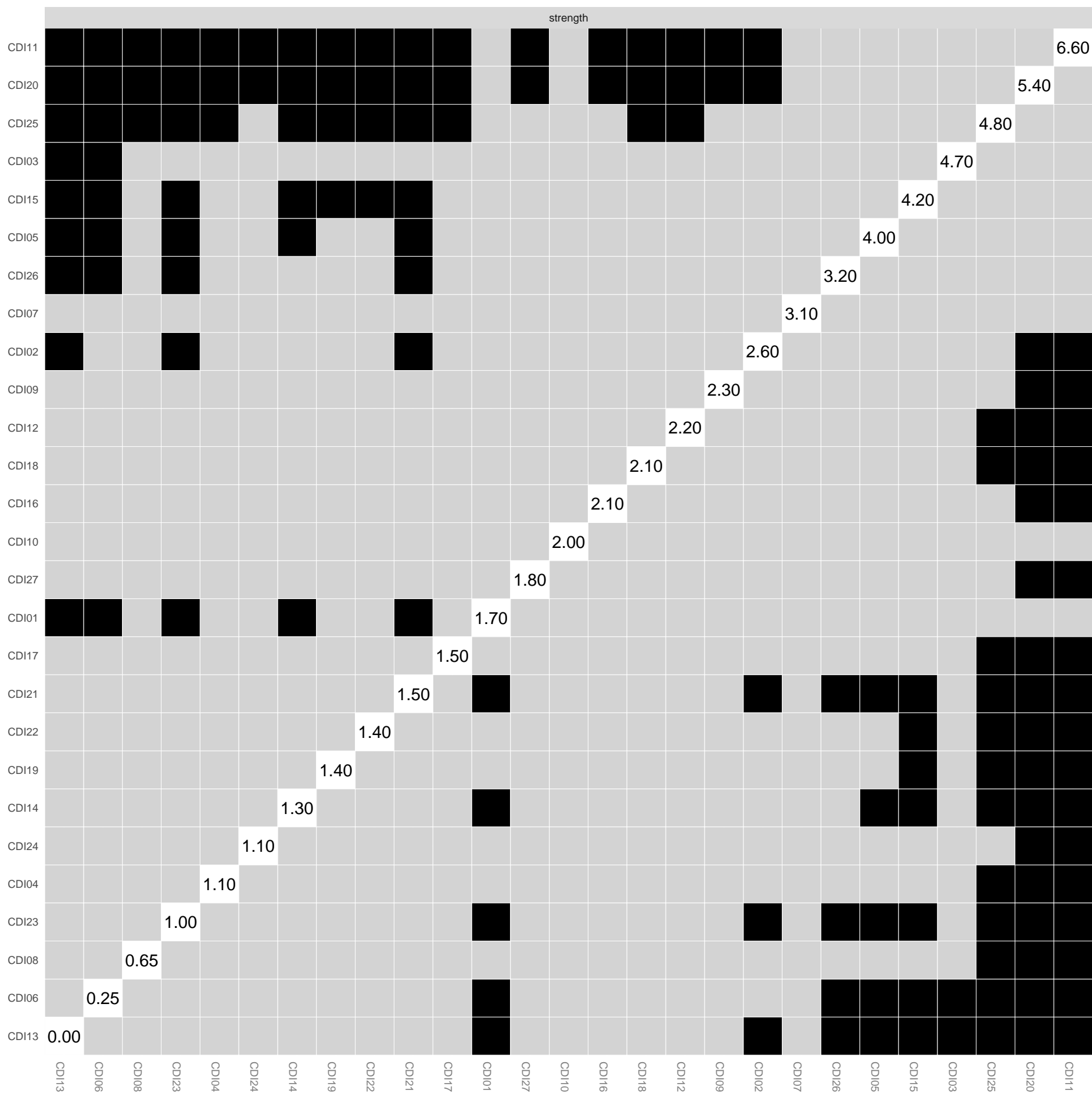

Supplement: Supplementary file 4 — Figure S4: Nonparametric bootstrapped difference test for node strength in the CDI‐P network. Note: Gray boxes indicate node strength that do not differ significantly from one another, while black boxes indicate node strength that do differ significantly. The numbers in the white boxes (i.e., diagonal line) represent the values of node strength. CDI01 = Sadness; CDI02 = Pessimism; CDI03 = Self‐Deprecation; CDI04 = Anhedonia; CDI05 = Misbehavior; CDI06 = Pessimistic Worrying; CDI07 = Self‐Hatred; CDI08 = Self‐Blame; CDI09 = Suicidal Ideation; CDI10 = Crying; CDI11 = Irritability; CDI12 = Social Withdrawal; CDI13 = Indecisiveness; CDI14 = Negative Body Image; CDI15 = School Work Difficulty; CDI16 = Sleep Disturbance; CDI17 = Fatigue; CDI18 = Reduced Appetite; CDI19 = Somatic Concerns; CDI20 = Loneliness; CDI21 = School Dislike; CDI22 = Lack of Friendship; CDI23 = School Performance Decrement; CDI24 = Low Self‐Esteem; CDI25 = Feeling Unloved; CDI26 = Disobedience; CDI27 = Fights. [file PCHJ-14-685-s009.pdf]

Average correlation with original sample

strength

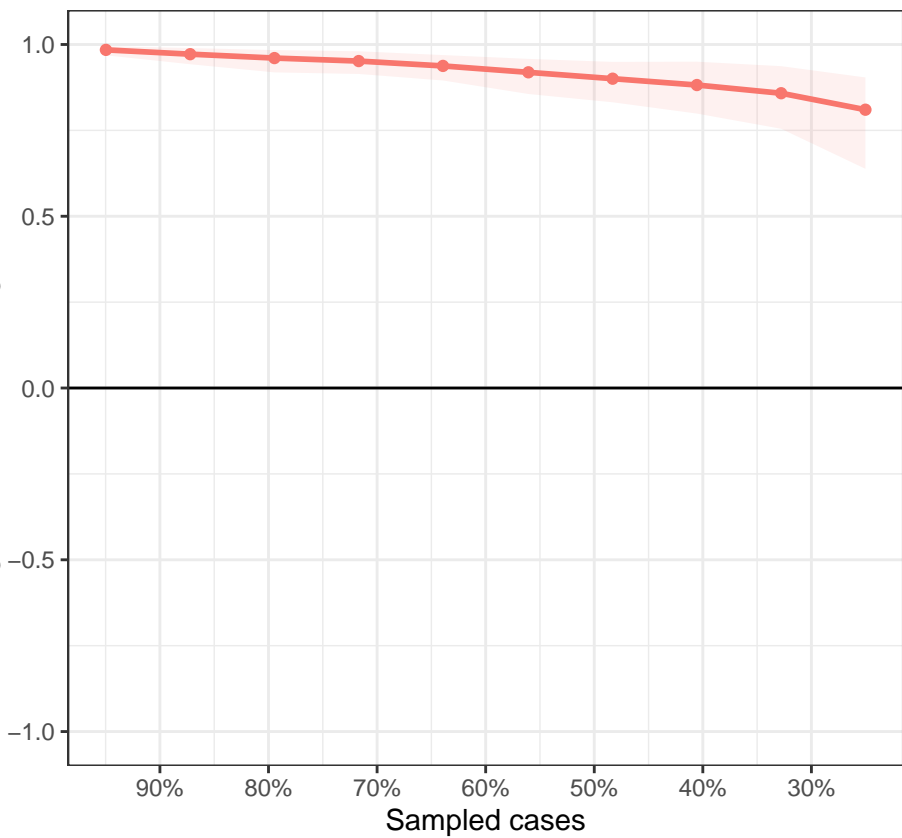

Supplement: Supplementary file 5 — Figure S5: Stability of node strength in the CDI‐Y network. Note: The x‐axis represents the percentage of cases in the original sample used at each step. The y‐axis represents the average of correlations between the centrality indices in the original network and the centrality indices in the networks that were re‐estimated after dropping increasing percentages of cases. [file PCHJ-14-685-s007.pdf]

● Bootstrap mean    ● Sample

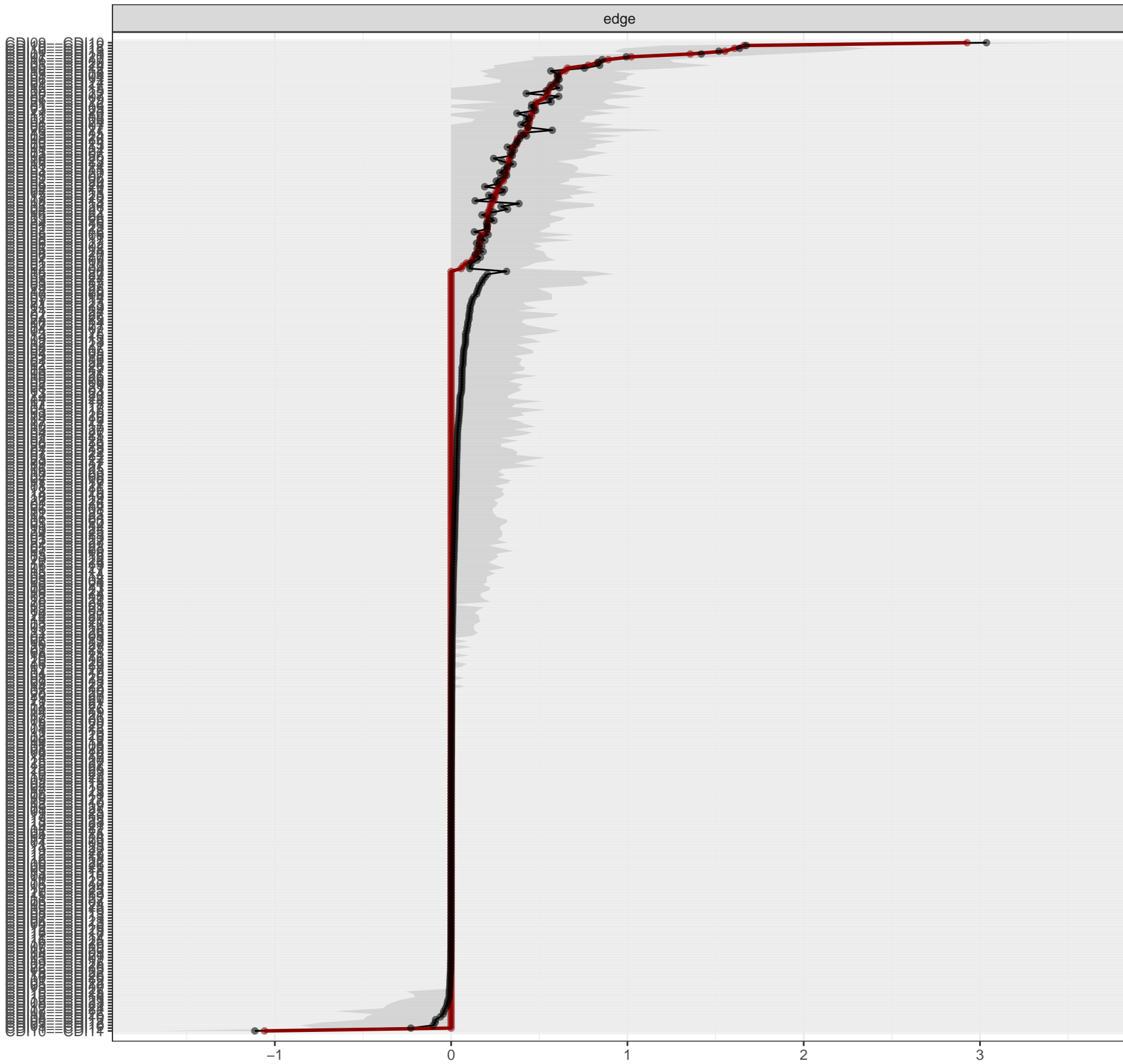

Supplement: Supplementary file 6 — Figure S6: Accuracy of edge weights in the CDI‐Y network. Note: The red line depicts the sample edge weights and the gray bar depicts the bootstrapped confidence interval. CDI01 = Sadness; CDI02 = Pessimism; CDI03 = Self‐Deprecation; CDI04 = Anhedonia; CDI05 = Misbehavior; CDI06 = Pessimistic Worrying; CDI07 = Self‐Hatred; CDI08 = Self‐Blame; CDI09 = Suicidal Ideation; CDI10 = Crying; CDI11 = Irritability; CDI12 = Social Withdrawal; CDI13 = Indecisiveness; CDI14 = Negative Body Image; CDI15 = School Work Difficulty; CDI16 = Sleep Disturbance; CDI17 = Fatigue; CDI18 = Reduced Appetite; CDI19 = Somatic Concerns; CDI20 = Loneliness; CDI21 = School Dislike; CDI22 = Lack of Friendship; CDI23 = School Performance Decrement; CDI24 = Low Self‐Esteem; CDI25 = Feeling Unloved; CDI26 = Disobedience; CDI27 = Fights. [file PCHJ-14-685-s002.pdf]

Average correlation with original sample

strength

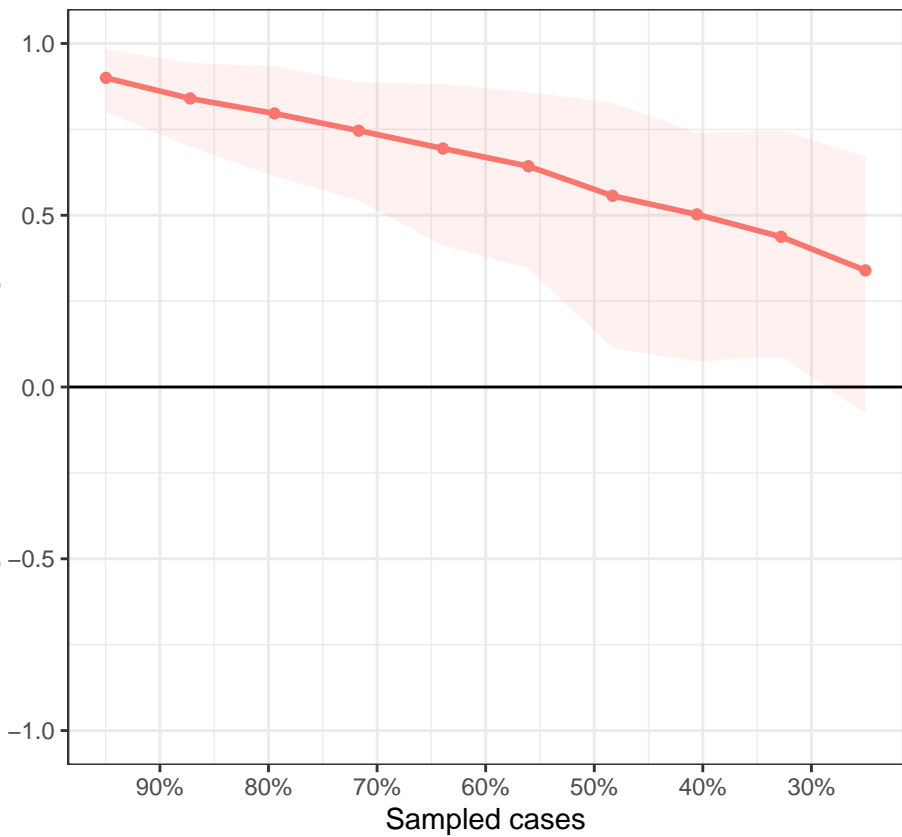

Supplement: Supplementary file 7 — Figure S7: Stability of node strength in the CDI‐P network. Note: The x‐axis represents the percentage of cases in the original sample used at each step. The y‐axis represents the average of correlations between the centrality indices in the original network and the centrality indices in the networks that were re‐estimated after dropping increasing percentages of cases. [file PCHJ-14-685-s003.pdf]

● Bootstrap mean ● Sample

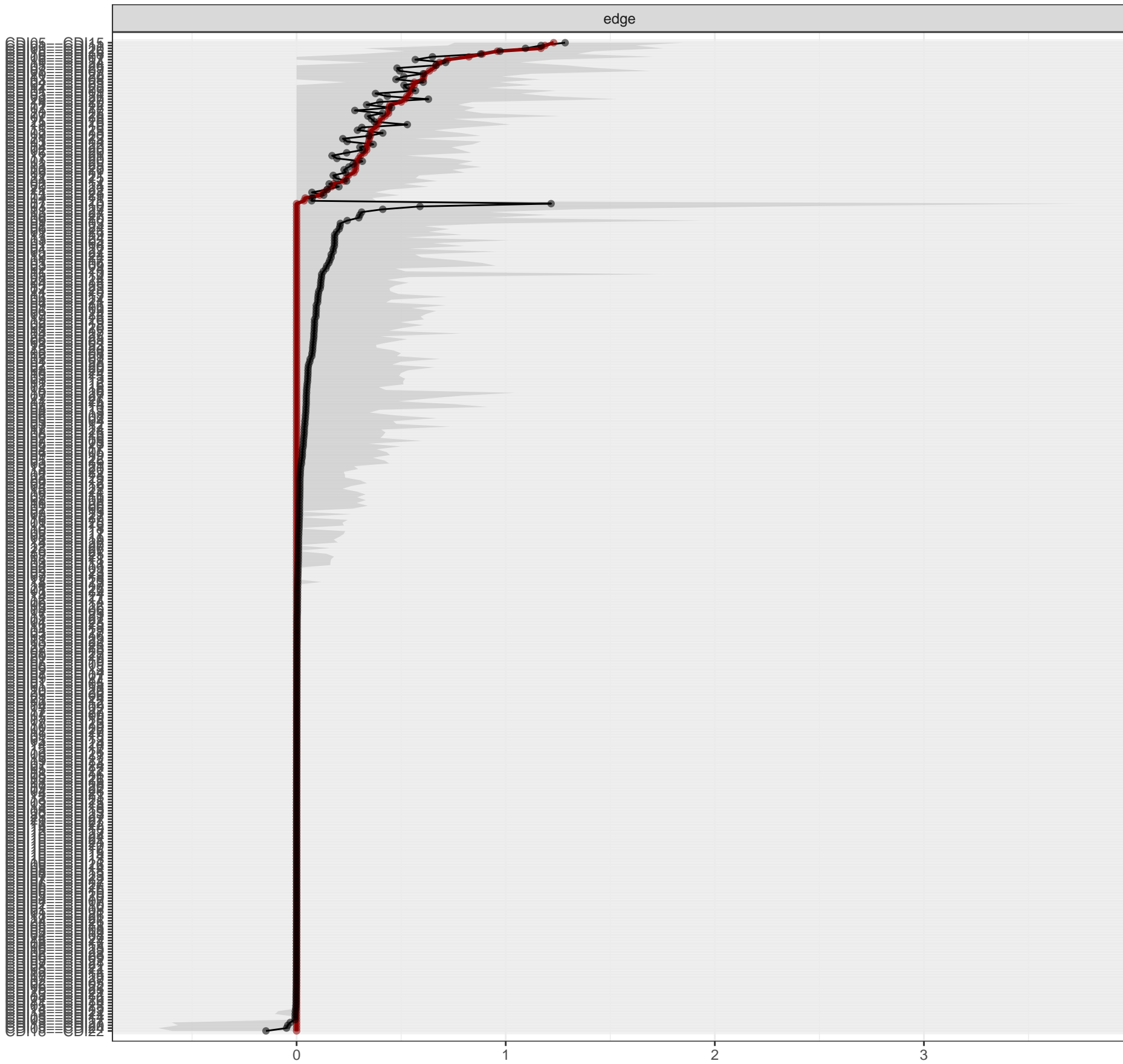

Supplement: Supplementary file 8 — Figure S8: Accuracy of edge weights in the CDI‐P network. Note: The red line depicts the sample edge weights and the gray bar depicts the bootstrapped confidence interval. CDI01 = Sadness; CDI02 = Pessimism; CDI03 = Self‐Deprecation; CDI04 = Anhedonia; CDI05 = Misbehavior; CDI06 = Pessimistic Worrying; CDI07 = Self‐Hatred; CDI08 = Self‐Blame; CDI09 = Suicidal Ideation; CDI10 = Crying; CDI11 = Irritability; CDI12 = Social Withdrawal; CDI13 = Indecisiveness; CDI14 = Negative Body Image; CDI15 = School Work Difficulty; CDI16 = Sleep Disturbance; CDI17 = Fatigue; CDI18 = Reduced Appetite; CDI19 = Somatic Concerns; CDI20 = Loneliness; CDI21 = School Dislike; CDI22 = Lack of Friendship; CDI23 = School Performance Decrement; CDI24 = Low Self‐Esteem; CDI25 = Feeling Unloved; CDI26 = Disobedience; CDI27 = Fights. [file PCHJ-14-685-s010.pdf]
