# Supplementary material for: Cross‐Informant Comparison of Depressive Symptoms in Youth: A Network Approach
Source: Psych J. 2025 Sep 2;14(5):685–96. doi: 10.1002/pchj.70050 (PMC12520835; doi:10.1002/pchj.70050)
Supplement: Supplementary file 3 — Figure S3: Nonparametric bootstrapped difference test for node strength in the CDI‐Y network. Note: Gray boxes indicate node strength that do not differ significantly from one another, while black boxes indicate node strength that do differ significantly. The numbers in the white boxes (i.e., diagonal line) represent the values of node strength. CDI01 = Sadness; CDI02 = Pessimism; CDI03 = Self‐Deprecation; CDI04 = Anhedonia; CDI05 = Misbehavior; CDI06 = Pessimistic Worrying; CDI07 = Self‐Hatred; CDI08 = Self‐Blame; CDI09 = Suicidal Ideation; CDI10 = Crying; CDI11 = Irritability; CDI12 = Social Withdrawal; CDI13 = Indecisiveness; CDI14 = Negative Body Image; CDI15 = School Work Difficulty; CDI16 = Sleep Disturbance; CDI17 = Fatigue; CDI18 = Reduced Appetite; CDI19 = Somatic Concerns; CDI20 = Loneliness; CDI21 = School Dislike; CDI22 = Lack of Friendship; CDI23 = School Performance Decrement; CDI24 = Low Self‐Esteem; CDI25 = Feeling Unloved; CDI26 = Disobedience; CDI27 = Fights. [file PCHJ-14-685-s004.pdf]

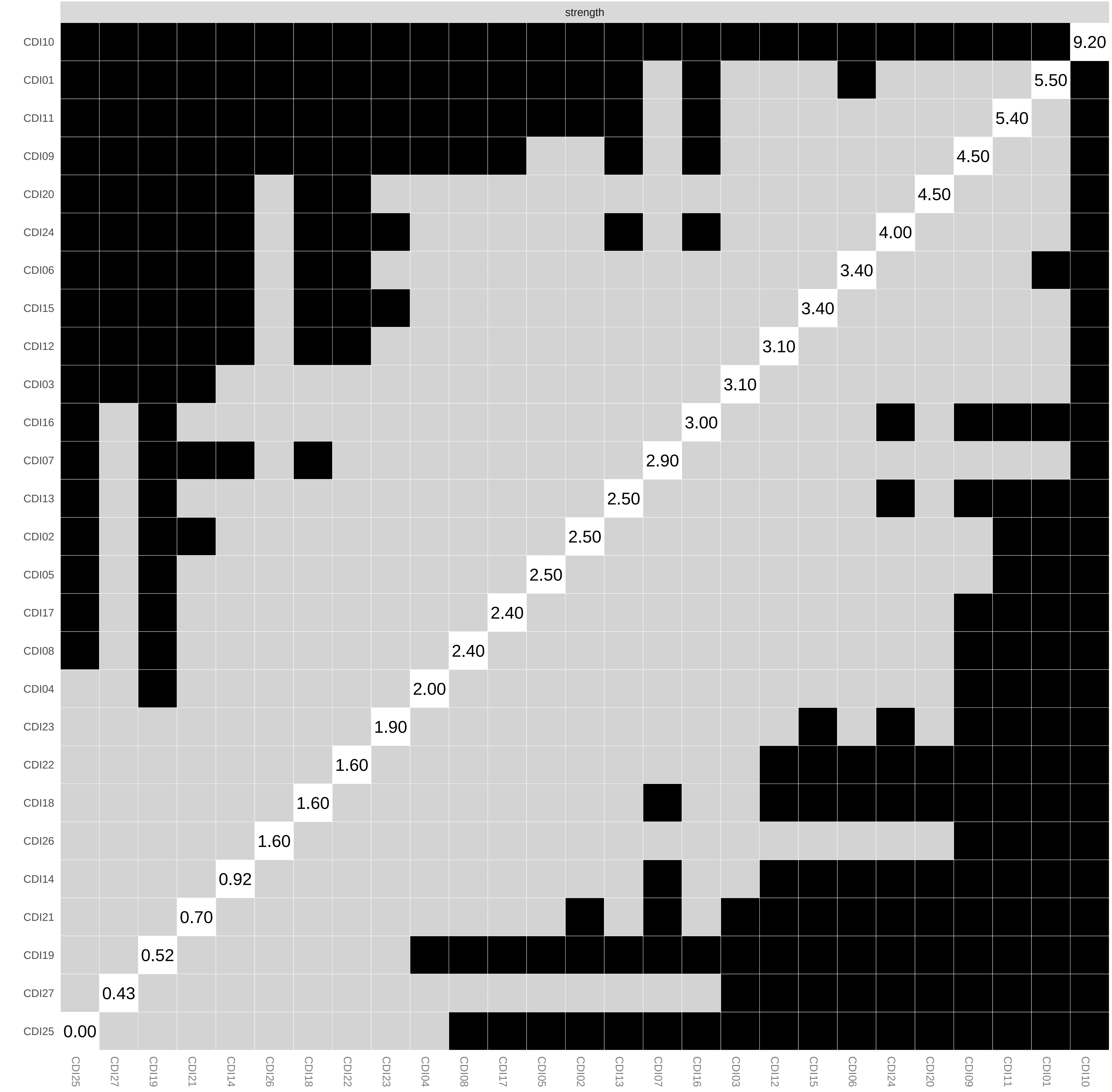
strength
CDI10
CDI01
CDI11
CDI09
CDI20
CDI24
CDI06
CDI15
CDI12
CDI03
CDI16
CDI07
CDI13
CDI02
CDI05
CDI17
CDI08
CDI04
CDI23
CDI22
CDI18
CDI26
CDI14
CDI21
CDI19
CDI27
CDI25
9.20
5.50
5.40
4.50
4.50
4.00
3.40
3.40
3.10
3.10
3.00
2.90
2.50
2.50
2.50
2.40
2.40
2.00
1.90
1.60
1.60
1.60
0.92
0.70
0.52
0.43
0.00
CDI25
CDI27
CDI19
CDI21
CDI14
CDI26
CDI18
CDI22
CDI23
CDI04
CDI08
CDI17
CDI05
CDI02
CDI13
CDI07
CDI16
CDI03
CDI12
CDI15
CDI06
CDI24
CDI20
CDI09
CDI11
CDI01
CDI10
